# Maternal survival in a low-resource setting, Mpilo Central Hospital, Bulawayo, Zimbabwe

**DOI:** 10.1186/s13104-020-4911-y

**Published:** 2020-01-30

**Authors:** Solwayo Ngwenya, Faith Nleya, Desmond Mwembe

**Affiliations:** 1 Mpilo Central Hospital, P.O. Box 2096, Vera Road, Mzilikazi, Bulawayo, Matabeleland Zimbabwe; 2 Royal Women’s Clinic, 52A Cecil Avenue, Hillside, Bulawayo, Zimbabwe; 3 National University of Science and Technology Medical School, Bulawayo, Matabeleland Zimbabwe; 4 National University of Science & Technology, P. O. Box AC 939, Ascot, Bulawayo, Zimbabwe

**Keywords:** Maternal mortality, Complications, Survival times, Safe motherhood, Low-resource settings

## Abstract

**Objectives:**

Maternal mortality is an important global subject. This dataset was generated from a retrospective cross-sectional study carried out at Mpilo Central Hospital, covering the period January 1, 2015 to December 31, 2018. The aim of the study was to compare how frequently the exposure to a risk factor was related to maternal death. Maternal deaths that were recorded during the study period were considered as cases. Controls were selected randomly from women of child-bearing age who survived during the study period. Low-resourced countries contribute significantly to global maternal deaths. Understanding risk factors could help reduce maternal mortality.

**Data description:**

The dataset contains data of 387 pregnant women who were included in the study. Data were collected as secondary data using a data collection sheet, as recorded by the hospital staff that gave all necessary demographic details in birth and mortality registers. The data collected included socio-demographic and clinical data. The independent variables were maternal age, gravidity, parity, antenatal visits, booking status, marital status, educational status, days spent in hospital, mode of delivery, fetal outcomes, and maternal complications. The dependent variable was maternal mortality. The data can be used to determine the relationship between the independent variables and maternal death.

## Objectives

Maternal mortality is of immense global interest. The leading causes of maternal deaths in 2017 were postpartum haemorrhage, sepsis and hypertensive disorders of pregnancy [1]. Low-resourced countries contribute significantly to global maternal deaths in Sub-Saharan Africa accounted for roughly two-thirds of maternal deaths.

Sub-Saharan Africa and Southern Asia accounted for 86% of the estimated global maternal deaths in 2017 [1]. The causes of maternal deaths are numerous and vary from one place to another depending on factors prevailing. The main direct causes of maternal death in developing countries include hemorrhage, sepsis, obstructed labor and hypertensive disorders [1, 2]. Maternal mortality occurring in high-income countries shows that maternal mortality is preventable. This dataset was generated from a retrospective cross-sectional study carried out at Mpilo Central Hospital, covering the period January 1, 2015 to December 31, 2018. Mpilo Central Hospital is government tertiary hospital, located in the township of Mzilikazi in Bulawayo. Bulawayo, located in Matabeleland is the second largest city in Zimbabwe after the capital city Harare. The aim of the study was to compare how frequently the exposure to a risk factor was related to maternal death. The study population for this study was all women of child-bearing age, aged between 15 and 49 years who died during the study period. Maternal deaths that were recorded during the study period were considered as cases. Controls were selected randomly from women of child-bearing age who survived during the same study period.

## Data description

The data were collected as secondary data using a data collection sheet, as recorded by the hospital staff that gave all necessary demographic details in birth and mortality registers. The data collected included variables such as age, gestational age, gravidity, parity, past obstetric history, mode of delivery, level of education, booking status, number of antenatal visits, perinatal outcomes, marital status, maternal mortality and days spent in the hospital. The dataset contains data of 387 women, aged between 15 and 49 years who died during the study period.

Data were manually collected from paper records and entered into the Microsoft Excel spreadsheet which can then be exported to Stata 12.1 statistical package for analysis. Descriptive statistics can be performed and presented as frequencies and percentages for categorical variables. Variance Inflated Factor (VIF) can be applied to test for multi-collinearity for all the candidate explanatory variables.

Binary logistic regression can be used to calculate the probability of maternal death given different variables. The Hosmer–Lemeshow test can be used to check the goodness of fit of the model, 95% Confidence Intervals (CI). The goodness of fit shows how well the data fits the model. Backward elimination can be used. A p-value < 0.05 can be taken as statistically significant.

The Cox proportional hazard model can be used on the factors that are statistically significant associated with maternal mortality to check if they have an effect on the survival time of patients. A p-value of < 0.0001 can be used to identify if the model fits the data and can be used to predict survival. Kaplan–Meier survival curves can be used to compare survival of patients with respect to their booking status.

Table 1 provides an overview of all data files/data sets described in this Data note. The Data can be accessed on Mendeley Data at http://dx.doi.org/10.17632/5gp976kchf.1 [3]. There is also a variables table in the dataset collected.

**Table 1:** Overview of data files/data sets

Label	Name of data file/data set	File types (file extension)	Data repository and identifier (DOI or accession number)
Dataset file 1 [3]	Matdata	Excel	DOI: http://dx.doi.org/10.17632/5gp976kchf.1.

## Limitations

The main limitations of the dataset are that the dataset contains few variables, and could have been included other exploratory variables like therapeutic interventions that could affect survival. The data was also secondary data collected retrospectively that could have caused some incorrect data being collected.

## Data Availability

The data described in this Data note can be freely and openly accessed on Mendeley Data at http://dx.doi.org/10.17632/5gp976kchf.1 [3]. Please see Table 1 and reference list for details and links to the data.

## Abbreviations

CI: confidence interval
VIF: Variance Inflated Factor
